# Tumor suppression effect of ultrasound-sensitive nanoparticles with focused ultrasound in a pancreas cancer xenograft model

**DOI:** 10.1186/s41747-024-00436-2

**Published:** 2024-03-20

**Authors:** Soojin Kim, Jae Young Lee, Eun-Joo Park, Yun Deok Ahn, Yuri Cheon, Wonchul Sim, Hak Jong Lee

**Affiliations:** 1 Department of Radiology, Seoul National University Hospital, Seoul, Republic of Korea; 2 Department of Radiology, Seoul National University College of Medicine, 103 Daehak-Ro, Jongno-Gu, Seoul, Republic of Korea 03080; 3 Biomedical Research Institute, Seoul National University Hospital, Seoul, Republic of Korea; 4 IMGT Company, Ltd, Seongnam, Republic of Korea; 5 Department of Radiology, Seoul National University Bundang Hospital, Seongnam, Republic of Korea; 6 Department of Medical Device Development, Seoul National University College of Medicine, Seoul, Republic of Korea

**Keywords:** Doxorubicin, Heterografts, Liposomal doxorubicin, Pancreatic neoplasms, Ultrasonic therapy

## Abstract

**Background:**

We investigated the tumor suppression effect of an ultrasound-sensitive doxorubicin-loaded liposome-based nanoparticle, IMP301, to enhance the synergistic effect with focused ultrasound (FUS) in an animal model of pancreatic cancer.

**Methods:**

Thirty nude mice with xenografts of PANC-1 human pancreatic cancer cells were randomly and prospectively allocated to 6 different groups (5 per group) each for Study-1 (dose–response test) and Study-2 (synergistic effect test). Study-1 consisted of control, gemcitabine, Doxil with FUS, and three different doses of IMP301 (2, 4, 6 mg/kg) with FUS groups. Study-2 consisted of control, FUS only, gemcitabine, Doxil with FUS, and IMP301 (4 mg/kg) with or without FUS groups. Differences in tumor volume and growth rate were evaluated by one-way ANOVA and Student–Newman–Keuls test.

**Results:**

In Study-1, 4 mg/kg or greater IMP301 with FUS groups showed lower tumor growth rates of 14 ± 4 mm^3^/day (mean ± standard deviation) or less, compared to the control, gemcitabine, and Doxil with FUS groups with rates exceeding 28 ± 5 (*p* < 0.050). The addition of FUS in Study-2 decreased the tumor growth rate in the IMP301-treated groups from 36 ± 17 to 9 ± 6, which was lower than the control, FUS only, gemcitabine, and Doxil with FUS groups (*p* < 0.050).

**Conclusions:**

IMP301 combined with FUS exhibited higher tumor growth suppression compared to the use of a conventional drug alone or the combination with FUS. The present study showed the potential of IMP301 to enhance the synergistic effect with FUS for the treatment of pancreatic cancer.

**Relevance statement:**

This article aims to evaluate the synergistic effect of FUS and ultrasound-responsive liposomal drug in tumor growth suppression by using xenograft mouse model of pancreatic ductal adenocarcinoma. FUS-induced ultrasound-sensitive drug release may be a potential noninvasive repeatable treatment option for patients with locally advanced or unresectable pancreatic cancer.

**Key points:**

• Modification of conventional drugs combined with FUS would maximize tumor suppression.

• IMP301 with FUS had higher tumor suppression effect compared to conventional chemotherapy.

• This image-guided drug delivery would enhance therapeutic effects of systemic chemotherapy.

**Graphical Abstract:**

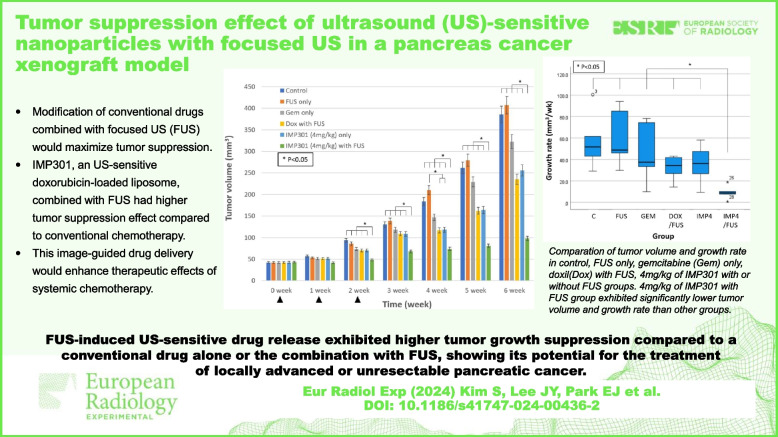

**Supplementary Information:**

The online version contains supplementary material available at 10.1186/s41747-024-00436-2.

## Background

Focused ultrasound (FUS) is a noninvasive therapeutic modality that uses ultrasound of higher intensity than diagnostic ultrasound concentrated only into a volume of interest to exert a site-specific therapeutic effect. FUS exerts thermal or nonthermal/mechanical effects on tissues depending on intensity, frequency, and exposure conditions, which may be modified based on the desired effect. The effectiveness of FUS has been demonstrated in the treatment of various tumorous or nontumorous diseases, including essential tremor, uterine fibroids, and prostate cancer [1]. FUS kills cancer cells using thermal ablation, mechanical disruption, or enhancement of drug delivery via hyperthermia or mechanical stimulation.

Pancreatic ductal adenocarcinomas tend to be diagnosed at a late stage, and only 15−20% of cases are considered potentially resectable at the time of initial diagnosis [2]. For most patients, chemotherapy is the only treatment option [3]. The dense fibrotic tissue of pancreatic cancer complicates drug penetration and hinders the development of effective targeted treatment. Many attempts have been made to improve the efficacy of targeted drug delivery into cancer cells using diverse modalities, including pH, temperature, and FUS [4].

Among the various types of drug delivery systems, FUS has the advantages of enabling real-time localization and fine modulation by controlling physical parameters. Mechanical effect-dominant FUS, which is characterized by high intensity and a short duty cycle, is preferred in drug delivery because it maximizes the mechanical effect and minimizes the thermal damage to the targeted tissue [5, 6]. A previous study showed that a doxorubicin-loaded microparticle–microbubble complex together with mechanical effect-dominant FUS conditions showed a higher tumor suppressive effect in a pancreatic cancer xenograft model than conventional intravenous drug delivery by increasing the intracellular uptake of chemotherapeutic drugs [7].

Based on the need for a stabilized and effective ultrasound-sensitive drug delivery system, we developed an ultrasound-sensitive liposome-based doxorubicin HCl-loaded nanoparticle, named IMP301, that responded to FUS itself and released drugs without the aid of microbubbles or other additives [8]. It was designed to optimize the stability of lipid membranes to prevent off-target side effects caused by indiscriminate release of drugs and allow sufficient on-target drug release via the formation of FUS-induced small pores [9].

In this prospective study, we conducted two distinct investigations to assess the therapeutic effect of IMP301 in combination with FUS on pancreatic cancer using a mouse xenograft model. The first phase, denoted as Study-1 or the dose–response test, aimed to establish the effective dose of IMP301 when used in conjunction with FUS, ensuring optimal therapeutic outcomes while minimizing adverse effects. Subsequently, Study-2, referred to as the synergistic effect test, was designed to validate and characterize the synergistic effects observed when combining IMP301 and FUS. We aimed to ensure the safety and efficacy of the combined intervention by this prospective dual-phase approach.

## Methods

The Institutional Animal Care and Use Committee of the Clinical Research Institute of Seoul National University Hospital approved this study (IACUC 21–0071).

### Animal model

Six-week-old male BALB/c nude mice were used for the animal model. The PANC-1 human pancreatic cancer cell line (Korean Cell Line Bank) was cultured in Dulbecco’s modified Eagle’s medium (DMEM, Invitrogen, CA, USA) supplemented with 10% fetal bovine serum (FBS, WelGene Co., Gyeongsan, Republic of Korea) and 1% penicillin (WelGene Co.) after digestion with 0.25% trypsin (WelGene Co.) at 37 °C. A total of 5 × 10^6^ cells suspended in 0.2 mL of medium were subcutaneously inoculated in the right flank. After 4 weeks to allow tumors to grow, mice began treatment (injection of drugs or irradiation of FUS) at weight range of 22−25 g.

### Ultrasound-sensitive liposome encapsulating doxorubicin (IMP301)

**Fig. 1:**
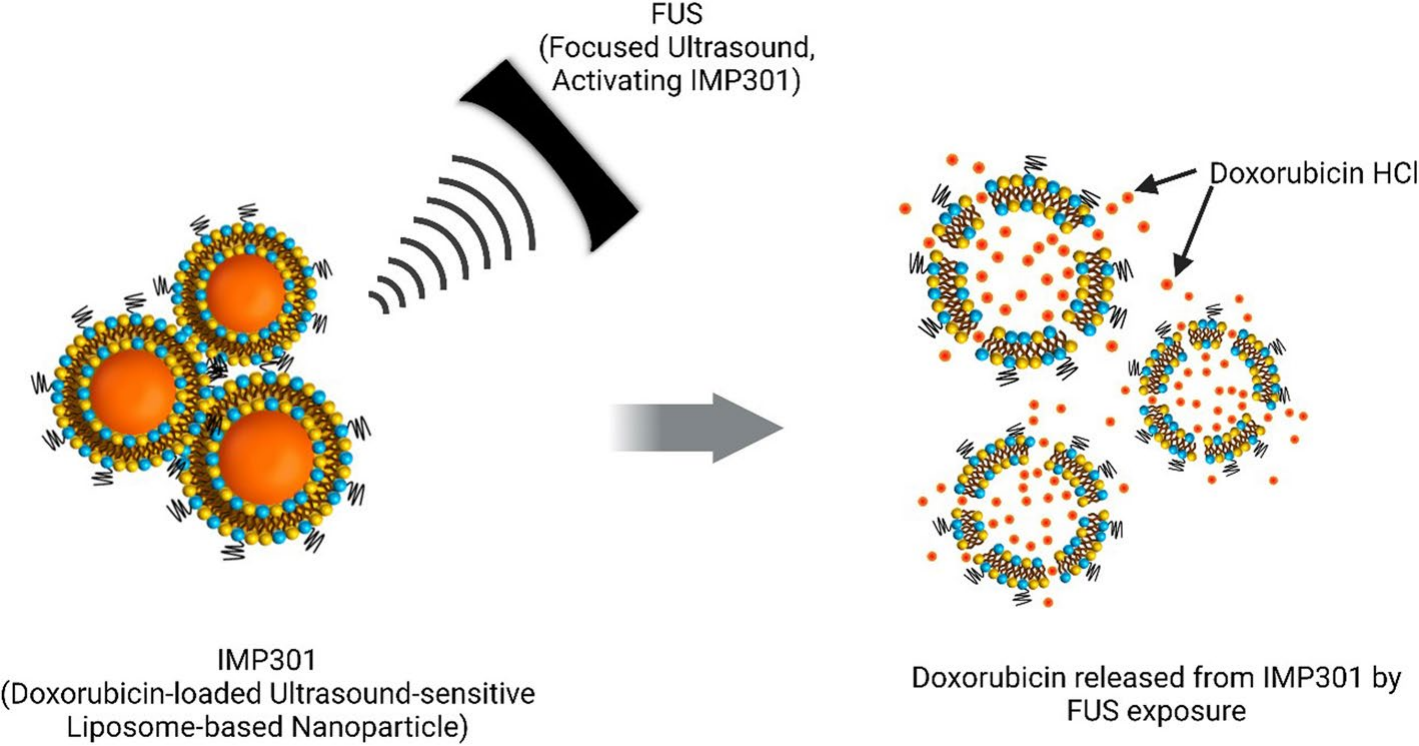
Mechanism of doxorubicin HCl release from IMP301. Schematic representation of IMP301, the ultrasound-sensitive liposome loaded with doxorubicin HCl. When exposed to the certain condition of FUS, the membrane of IMP301 is reconstructed and nanopores are formed in the lipid layer, then drugs are released from the carrier. *FUS* Focused ultrasound, *IMP301* Doxorubicin HCl-loaded liposome

The test particle IMP301, supplied by a drug-delivery platform company (IMGT Co., Seongnam, Republic of Korea), had its synthesis protocol detailed in previous studies [8, 10]. IMP301 liposomes, optimized for ultrasound sensitivity, were prepared using a mixture of distearoylphosphatidylcholine, distearoylphosphatidylcholine-polyethylene glycol, cholesterol, dioleoyl-sn-glycero phosphatidylethanolamine, and 1-myristoyl-2-stearoyl-sn-glycero-3-phosphocholine via an ethanol injection and extrusion method. Doxorubicin HCl was then encapsulated into the liposomes using the remote loading method, resulting in a final storage concentration of 2 mg/mL at 2–8 °C. When irradiated by unfocused continuous-wave ultrasound, IMP301 showed enhanced doxorubicin release compared to non-ultrasound-sensitive liposomal doxorubicin (Doxil) without a significant change in the size of liposomes (Supplementary Fig. S1). IMP301 also showed increased doxorubicin release compared to Doxil after FUS irradiation with high intensity low duty cycle conditions but not at low intensity high duty cycles with the same total energy (Supplementary Fig. S2). These findings indicate that doxorubicin was released from IMP301 due to ultrasound-mediated pore formation in liposome membranes, and it was dependent on the pressure of FUS instead of hyperthermia, as mentioned in previous studies [8, 9] (Fig. 1). Pharmacokinetic profiles of IMP301 compared to free doxorubicin or Doxil were demonstrated in the previous study [8]. *Ex vivo* release and intratumoral uptake of doxorubicin encapsulated in IMP301 compared to Doxil were provided in the previous study [8]. Biodistribution and *ex vivo* images of IMP301 showed that increased concentration and selective distribution of IMP301 within the tumor were noted under FUS exposure, compared to no exposure (Supplementary Fig. S3).

### FUS condition

FUS system had two transducers, each for imaging guidance and treatment. After placing the xenograft mouse on a three-dimensional positioning holder submerged in 36 °C degassed water, tumor was localized by the imaging-guidance transducer of FUS system [7]. Then, FUS was irradiated to the target area under ultrasound guidance using a pre-clinical therapeutic ultrasound system (VIFU2000 1 MHz, ALPINION Medical Co., Seoul, Republic of Korea). The optimal sonication conditions for effective drug release from IMP301 has been studied [8]. The following acoustic parameters were used: 2% duty cycle, 250-Hz pulse repetition frequency, 20 s/spot insonation time, 9.2 mm × 1.3 mm focal size length × diameter, 2 mm × 2 mm gap, and 72-W acoustic power. The duration of each focal spot was 20 s, and each tumor was irradiated by multiple focal spots to cover the entire tumor.

### Treatment protocol

The experiment was conducted from June to November, 2021, by two authors (Y.D.A., Y.C.). Gemcitabine (GEM) and pegylated liposomal doxorubicin, Doxil (DOX), were purchased from Boryung Pharmaceutical (Seoul, Republic of Korea) and Janssen Pharmaceuticals (Beerse, Belgium), respectively. Vehicle (dilution buffer without doxorubicin) was purchased from IMGT Co. (Seongnam, Republic of Korea).

PANC-1 xenografted mice were prepared for experiments and randomly, prospectively allocated to each study. Thirty mice were randomly assigned to 6 groups (5 for each group) in Study-1, as follows: (1) vehicle only control, (2) GEM 200 mg/kg only, (3) DOX 4 mg/kg with FUS, (4) IMP301 2 mg/kg with FUS, (5) IMP301 4 mg/kg with FUS, and (6) IMP301 6 mg/kg with FUS. The other 30 mice were randomly assigned to 6 groups (5 for each group) in Study-2, as follows: (1) vehicle only control, (2) vehicle with FUS, (3) GEM 200 mg/kg only, (4) DOX 4 mg/kg with FUS, (5) IMP301 4 mg/kg only, and (6) IMP301 4 mg/kg with FUS.

All animals were treated weekly for the first 3 weeks, and therapeutic agents were administered intravenously except GEM, which was administered intraperitoneally. All mice were maintained under general anesthesia during the treatment via an intraperitoneal injection of zolazepam hydrochloride (30 mg/kg, Zoletil, Virbac, Seoul, Korea) and xylazine hydrochloride (10 mg/kg, 2% Rompun, Bayer Korea, Seoul, Korea). After drug injection, the mice were placed under FUS system and FUS was performed after tumor localization without delay. Tumor volume and body weight were measured weekly until the 6th week.

The tumor volume was measured by ultrasound in three-dimension, using an L3-12H linear probe for animals (E-Cube, Alpinion, Seoul, Korea), and calculated as 1/2 × short length × long length × height (mm^3^). The tumor growth rate was calculated as (tumor volume at 6th week *minus* tumor volume at 0th week)/6 (mm^3^/week). The tumor growth rate since the 3rd week was calculated as (tumor volume at 6th week *minus* tumor volume at 3rd week)/3 (mm^3^/week).

### Statistical analysis

Representative values for tumor size and growth rate of each group are expressed as the means ± standard deviation. All statistical analyses were performed using one-way ANOVA and the post hoc Student–Newman–Keuls test to determine significant (*p* < 0.050) differences in tumor volume and growth rate. These tests were performed using SPSS Statistics 28.0.0.0 (IBM, New York, USA) by the one of the authors (S.K.).

## Results

### Study-1 (dose–response test)

**Fig. 2:**
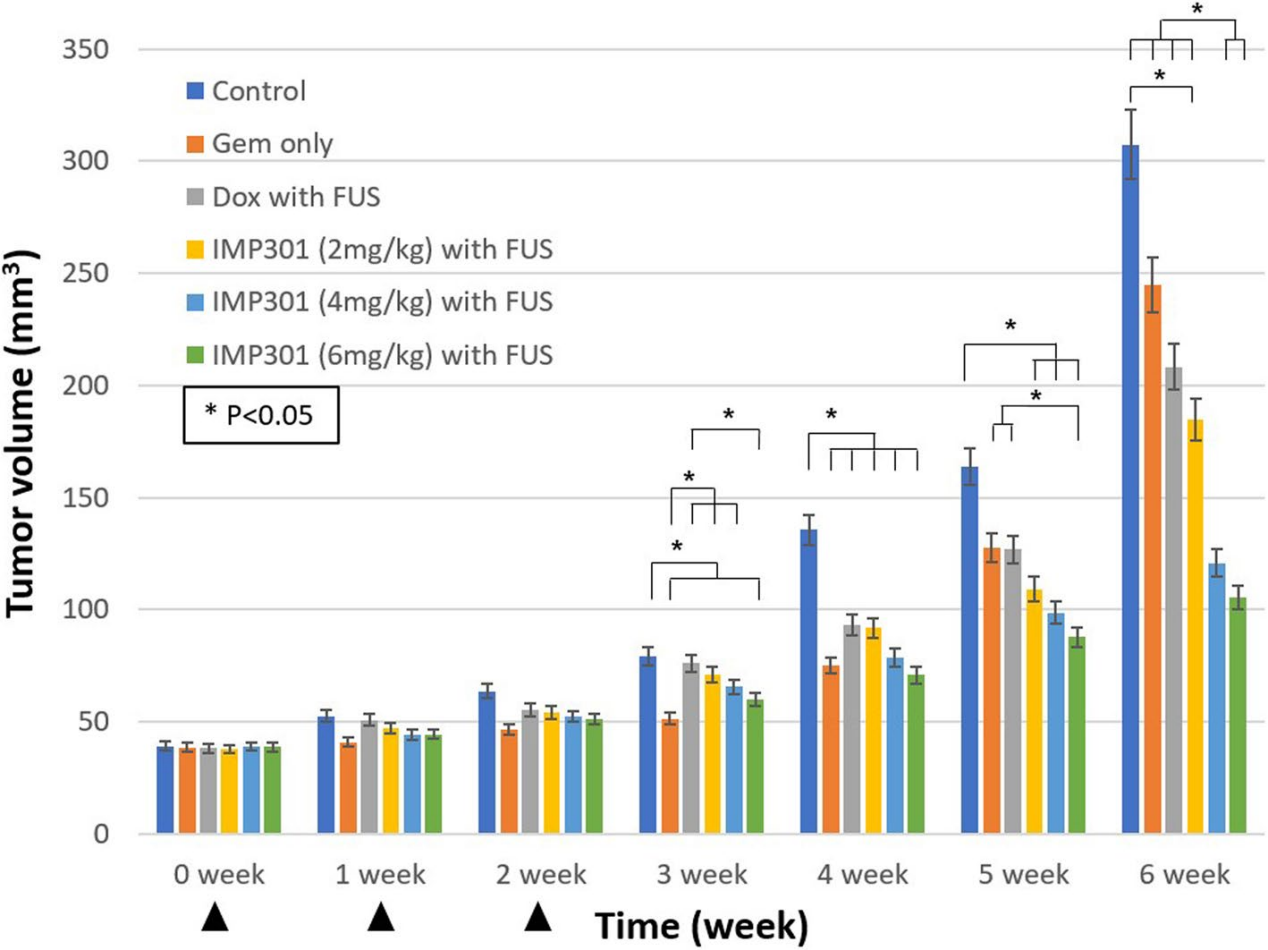
Tumor volume in dose response experiment. In the 5th and 6th week, the IMP301 6 mg/kg with FUS group showed the smallest tumor size, followed by the IMP301 4 mg/kg with FUS group. In the 5th week, at doses 4 mg/kg or greater, the IMP301 with FUS group had significantly lower tumor volume compared to control group (*p* < 0.050; statistical significance indicated as asterisk, *). Arrowheads indicate treatments. *DOX* Doxil, *FUS *Focused ultrasound, *GEM* Gemcitabine, *IMP301* Doxorubicin HCl-loaded liposome

**Fig. 3:**
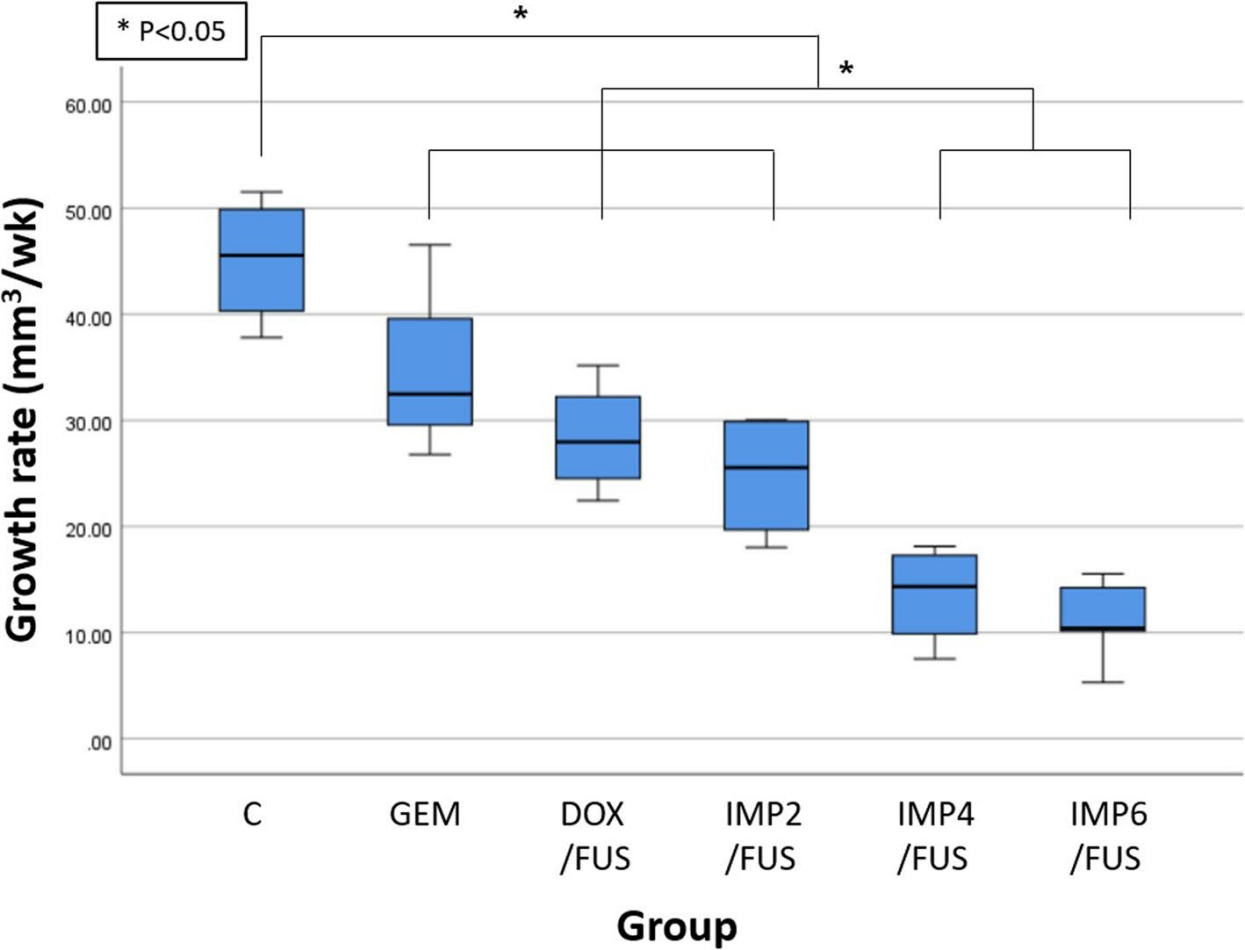
Tumor growth rate in dose-response experiment. At doses 4 mg/kg or greater, the IMP301 with FUS group had significantly lower tumor growth rate, compared to the control, GEM, DOX with FUS, and 2 mg/kg IMP301 with FUS groups (*p* < 0.050; statistical significance indicated as asterisk, *). There was no significant difference between tumor growth rate among GEM, DOX with FUS, and 2 mg/kg IMP301 with FUS groups, but all three groups showed lower tumor growth rate than the control group (*p* < 0.050). *DOX* Doxil, *FUS* Focused ultrasound, *GEM* Gemcitabine, *IMP301* Doxorubicin HCl-loaded liposome, *IMP2* 2 mg/kg IMP301, *IMP4* 4 mg/kg IMP301, *IMP6* 6 mg/kg IMP301, *C* Control

Among 30 male PANC-1 xenograft mice, during the 4th to the 6th weeks, one mouse each from the control, DOX with FUS, and two doses of IMP301 (2, 4 mg/kg) with FUS groups died and were excluded from the analyses. At the 6th week, the IMP301 with FUS group at doses 4 mg/kg or greater showed significantly lower average tumor volume compared to the control, GEM only, and DOX with FUS groups, but the GEM only and DOX with FUS groups did not show significant differences from the control group (*p* < 0.050) (Table 1, Fig. 2). IMP301 with FUS showed significantly lower tumor growth rate at doses 4 mg/kg or greater compared to the control, GEM, DOX with FUS, and 2mg/kg IMP301 with FUS groups (*p* < 0.050) (Table 1, Fig. 3). There was no significant difference between tumor growth rate among GEM, DOX with FUS, and 2mg/kg IMP301 with FUS groups, but all three groups showed lower tumor growth rate than the control group (*p* < 0.050) (Table 1, Fig. 3). The tumor growth rate after treatment completion (*i.e.*, tumor growth rate since the 3rd week) was significantly lower in the 4 mg/kg or greater IMP301 with FUS groups compared to the other groups (*p* < 0.050) (Table 1).

**Table 1:** Tumor volume and growth rate of groups in study-1

Values	Control	GEM only	DOX with FUS	IMP301 (2 mg/kg) with FUS	IMP301 (4 mg/kg) with FUS	IMP301 (6 mg/kg) with FUS
Volume at 0 week (mm^3^)	37 ± 6	37 ± 4	38 ± 2	36 ± 6	39 ± 7	39 ± 3
Volume at 5th week (mm^3^)	164 ± 21	131 ± 14	127 ± 19	109 ± 16	98 ± 21	88 ± 18
Volume at 6th week (mm^3^)	307 ± 32	245 ± 48	208 ± 25	185 ± 35	121 ± 28	105 ± 22
Growth rate (mm^3^/week)	45 ± 5	35 ± 7	28 ± 5	25 ± 5	14 ± 4	11 ± 4
Growth rate since 3rd week (mm^3^/week)	67 ± 19	58 ± 18	45 ± 10	38 ± 12	18 ± 7	15 ± 7

Numbers indicate the means ± standard deviation. *DOX* Doxil, *FUS* Focused ultrasound, *GEM* Gemcitabine, *IMP301* Doxorubicin HCl-loaded liposome

There was no significant weight decrease in the IMP301-treated groups compared to the control or FUS only group (Supplementary Table S1). Skin irritation was observed in the mice treated with 6 mg/kg IMP301 and FUS. Because there was no significant difference in the average tumor growth rate between 4 mg/kg and 6 mg/kg IMP301 (Table 1), 4 mg/kg IMP301 was selected for Study-2 as the effective dose to avoid the potential side effects of the drug.

### Study-2 (synergistic effect test)

**Fig. 4:**
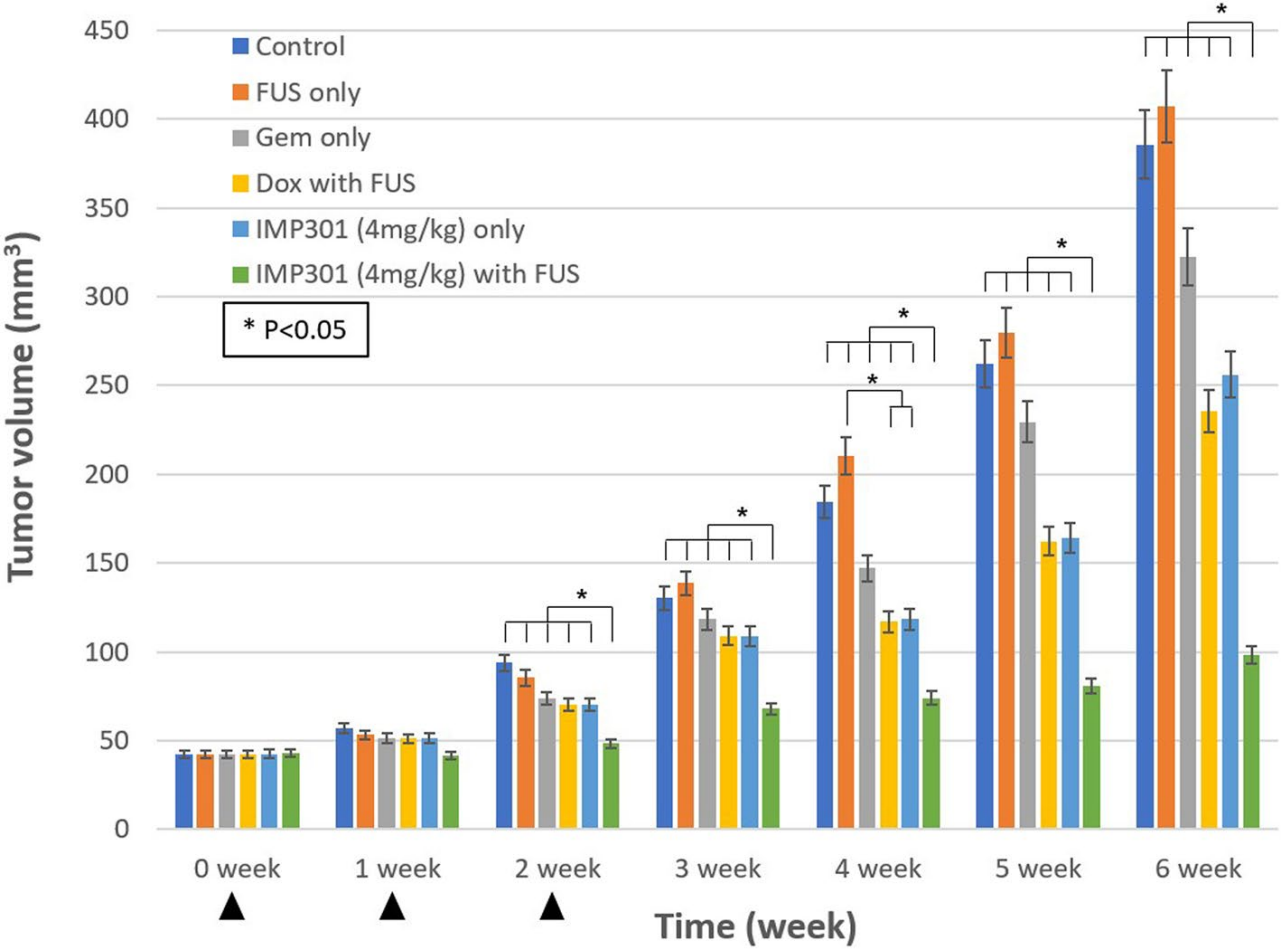
Tumor volume in synergistic effect experiment. In the 6th week, IMP301 4 mg/kg with FUS group was the only group which showed significantly lower tumor volume compared to the control group (*p* < 0.050; statistical significance indicated as asterisk, *). In addition, IMP301 4 mg/kg with FUS group showed lower tumor volume compared to Dox with FUS group, while IMP301 4 mg/kg only group did not show significant difference with Dox with FUS group (*p* < 0.050). Arrowheads indicate treatments. *DOX* Doxil, *FUS* Focused ultrasound, *GEM* Gemcitabine, *IMP301* Doxorubicin HCl-loaded liposome

**Fig. 5:**
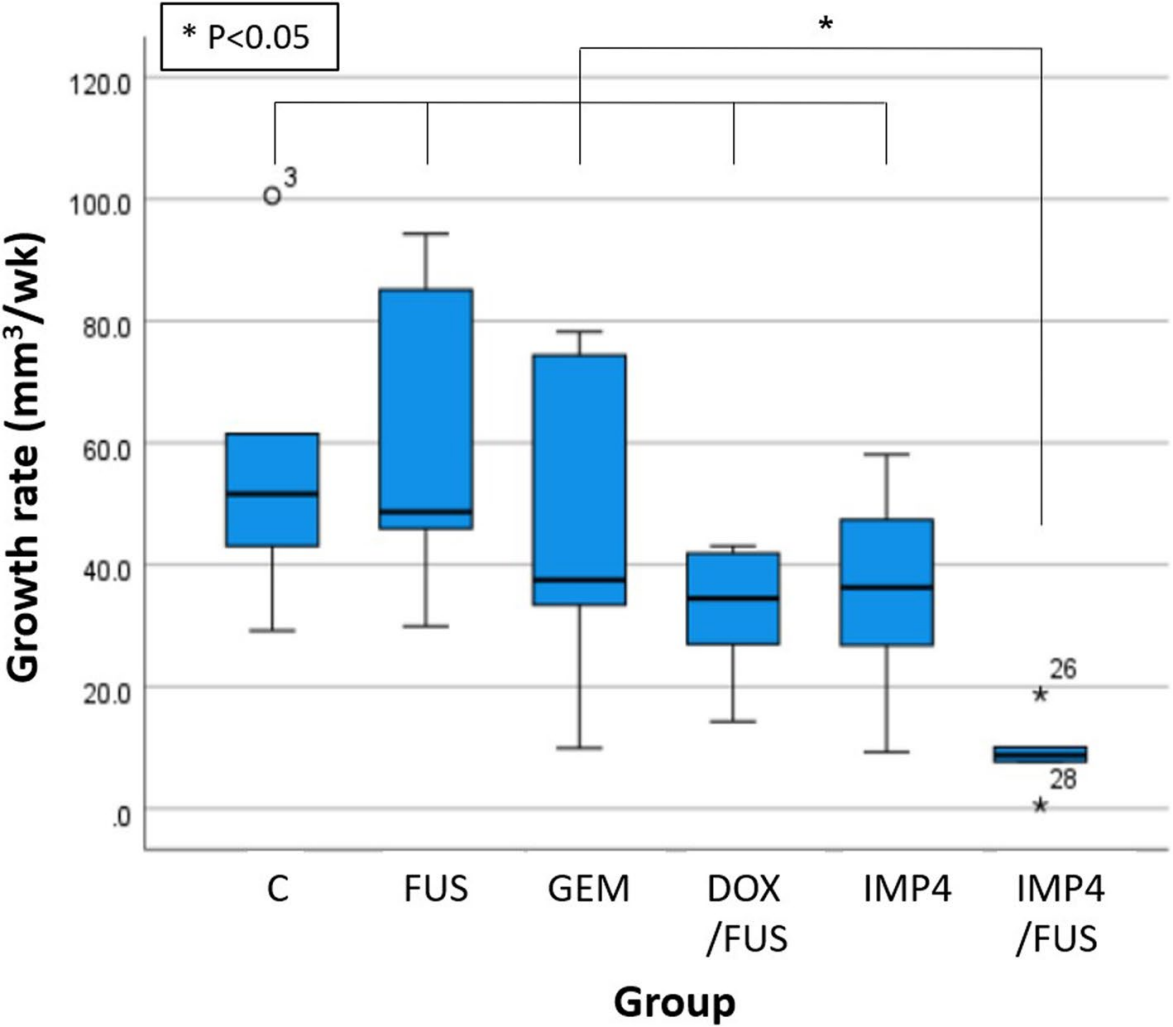
Tumor growth rate in synergistic effect experiment. Contrary to the IMP301 4 mg/kg only group, the IMP301 4 mg/kg with FUS group showed significantly lower tumor growth rate compared to all the other groups and the difference was only significant when irradiated with FUS (*p* < 0.050; statistical significance indicated as asterisk, *). *DOX* Doxil, *FUS* Focused ultrasound, *GEM* Gemcitabine, *IMP301* Doxorubicin HCl-loaded liposome, *IMP4* 4 mg/kg IMP301, *C *Control

Thirty male PANC-1 xenograft mice were tested and no mice died during the experiment. The IMP301 with FUS group showed a significantly lower average tumor volume in the 6th week compared to the other groups, including the control, FUS only, GEM only, DOX with FUS, and IMP301 only groups (*p* < 0.050), but the IMP301 only group did not show a significant difference from the other groups except the IMP301 with FUS group (Table 2, Fig. 4). The IMP301 with FUS group also exhibited a significantly lower tumor growth rate than the other groups (*p* < 0.050) (Table 2, Fig. 5). For the tumor growth rate after treatment completion (*i.e.*, tumor growth rate since the 3rd week), the IMP301 with FUS group showed the lowest value among the groups, with significant differences found between this group and both the control group, and the FUS only group (*p* < 0.050) (Table 2). The DOX with FUS group and IMP301 only group did not show any significant differences in tumor growth rate from the 3rd week compared with other groups. There were no significant differences in body weights between groups (Supplementary Table S2).

**Table 2:** Tumor volume and growth rate of groups in study-2

Values	Control	GEM only	FUS only	DOX with FUS	IMP 301 (4 mg/kg) only	IMP 301 (4 mg/kg) with FUS
Volume at 0 week (mm^3^)	42 ± 10	42 ± 8	42 ± 7	43 ± 7	43 ± 9	43 ± 10
Volume at 6th week (mm^3^)	386 ± 151	323 ± 158	407 ± 154	236 ± 60	256 ± 107	98 ± 42
Growth rate (mm^3^/week)	57 ± 24	47 ± 26	61 ± 25	32 ± 11	36 ± 17	9 ± 6
Growth rate since 3rd week (mm^3^/week)	85 ± 43	68 ± 47	89 ± 45	42 ± 16	49 ± 34	10 ± 11

Numbers indicate the means ± standard deviation. *DOX* Doxil, *FUS* Focused ultrasound, *GEM* Gemcitabine, *IMP301* Doxorubicin HCl-loaded liposome

## Discussion

This study demonstrated that the combination of IMP301 and FUS induced a significantly greater tumor growth suppression effect compared to conventional chemotherapeutic drugs, such as GEM and DOX. This result may be explained by the peritumoral release of the drug and mechanical stimulation to tumor tissues themselves by FUS. Peritumoral release might cause temporally higher local concentrations of the drug around the tumor, which likely led to higher penetration of the drug into the tumor [8]. The mechanical stimulation by FUS might cause the sonoporation of cells and microstreaming of extracellular fluid which likely increases cell permeability and drug influx as tumors experience higher positive and negative pressure.

This study suggests that ultrasound-sensitive liposome-based drugs developed by optimizing lipid components and ratios produced more tumor suppressive effects than conventional drugs when combined with FUS. FUS reaches the deepest part of our body and delivers mechanical energy when there is no air or bone in the pathway. Ultrasound-sensitive liposome-based agents with higher efficacy might become developed for any tumor in any location, including pancreatic cancer located deep within the abdomen. However, more research is needed in the future.

The combination of IMP301 and FUS also showed a persistent tumor suppression effect with a significantly lower tumor growth rate after completion of weekly treatment in the first 3 weeks, unlike GEM only or DOX with FUS treatment. Because there was no significant difference in tumor volume between groups, except the control when the weekly treatment was completed, FUS with IMP301 had a delayed effect, which was likely caused by apoptosis. It may be partially related to the long-term effect of maintaining a tumor-suppressive environment, as supported by previous studies [1, 11–13]. FUS may modulate the tumor microenvironment to become more immunogenic by enhancing the release of damage-associated molecular patterns and activating cytotoxic T cells against tumors by increased antigen presentation [14, 15]. However, this hypothesis needs further research.

We determined the experimental groups and excluded certain combinations based on ethical considerations and the objective of minimizing unnecessary sacrifices in our animal study. Specifically, GEM with FUS and DOX without FUS were excluded from the comparison groups. The exclusion of GEM with FUS was guided by our focus on evaluating the therapeutic efficacy of IMP301 and DOX when combined with FUS, as opposed to the well-established standard of care, GEM [16]. While acknowledging GEM’s status as a standard of care for pancreas cancer treatment, our study aimed to assess whether IMP301 with FUS could provide a comparable therapeutic effect [3]. Similarly, the decision to exclude DOX without FUS from our comparison groups was guided by our primary aim of evaluating the effects of FUS in combination with DOX and IMP301. While the inclusion of DOX without FUS could have yielded additional data, we opted to prioritize our study’s main objective, emphasizing the assessment of the combined therapeutic effects. This strategic decision aligns with the ethical consideration of minimizing the number of experimental conditions, ensuring a focused and substantiated analysis within the scope of our study design.

Although our study demonstrated therapeutic benefits of ultrasound-sensitive drugs enhanced with FUS, there are some limitations to overcome. First, we hypothesized that tumor volume represented cellularity and excluded the possibility of pseudo-progression. A few individuals showed an initial volume increase followed by a decrease until 6th week in both studies, which may be pseudo-progression. However, these outcomes did not affect the interpretation of the results because more cases of possible pseudo-progression were observed in the IMP301-treated groups than the other groups. Second, we did not consider tumor heterogeneity in treatment response within the same group. This factor may derive from the heterogeneity of the tumor itself, with increased sensitivity to certain drugs, which results in “outliers” with different growth patterns than other individuals within the same group. Although it did not affect statistical significance in this experiment, tumor heterogeneity must be considered in clinical settings for effective personalized treatment. Third, adverse effects of drugs other than body weight change were not fully evaluated. However, an increased risk of termination was not observed in this experiment. A previous study also showed that IMP301 with FUS did not result in a significant weight difference [8]. Although only body weight change was measured, there were no significant outcomes indicating increased adverse effects in IMP301-treated groups compared to conventional drugs, other than skin irritation at excessive doses. Fourth, there was a limitation in assessing the reproducibility of tumor volume. Tumor volumes were measured once for each animal, and the average within the same group was calculated at a certain period of time. We attempted to minimize measurement error by having the same person conduct all measurements. Fifth, we used subcutaneously inoculated pancreatic cancer in mice, which is different from an orthotopic tumor model. Clinical pancreatic cancer is surrounded by a dense fibrous stroma and hypoxic tumor microenvironment, which interfere with the desired delivery of drugs and penetration of ultrasound [17]. Its deep retroperitoneal location surrounded by adjacent organs also affect the irradiated ultrasound intensity. Future studies should be improved with an orthotopic model to better reflect the actual clinical setting and determine the optimal ultrasound conditions.

To summarize, our study demonstrated that IMP301 combined with short duty cycle pulsed FUS provided greater and persistent therapeutic effects in tumor suppression compared to conventional systemic chemotherapy in a pancreas cancer xenograft model in mice. FUS is a noninvasive repeatable targeted treatment modality that is available to patients with locally advanced disease or who are excluded from surgical indications. This image-guided drug delivery system may benefit patients by enhancing the therapeutic effects of systemically administered drugs and potential future applications in the treatment of other tumor types.

### Supplementary Information

**Additional file 1: Supplementary Fig. S1.** (a, b) Cumulative doxorubicin release from IMP301 or Doxil depending on unfocused continuous wave ultrasound irradiation. Both IMP301 and Doxil resulted in enhanced doxorubicin release when irradiated by unfocused ultrasound (continuous wave (100% duty cycle); 29 kHz frequency; 92 W/cm^2^ intensity). Cumulative release of doxorubicin from IMP301(a) was three-fold greater than that of Doxil (b) when irradiated by unfocused ultrasound. (c, d) Size distribution of liposomes depending on unfocused ultrasound irradiation time. Both IMP301 and Doxil liposomes maintained size throughout the experiment. *FUS* Focused ultrasound, *IMP301* doxorubicin HCl-loaded liposome, *US* ultrasound. **Supplementary Fig. S2.** (a) Doxorubicin release from IMP301 or Doxil by FUS exposure. IMP301 released more than two-fold amount of doxorubicin compared to Doxil when irradiated by FUS (2% duty cycle; 1.0-MHz frequency; 2.8 kW/cm^2^ intensity; 250-Hz pulse repetition frequency; 20 s/spot insonation time). (b) Doxorubicin release from IMP301 depending on the intensity and duty cycle of FUS. When exposed to FUS with same total amount of energy (70.8 W/cm^2^), doxorubicin release from IMP301 was mainly induced by burst mode of high-intensity beam rather than continuous mode of low-intensity beam. *FUS* Focused ultrasound, *IMP301* doxorubicin HCl-loaded liposome, *US* ultrasound. **Supplementary Fig. S3.** (a) Biodistribution of IMP301 dependent on FUS exposure. Increased IMP301 concentration and more selective distribution of IMP301 within the tumor were noted under FUS exposure, compared to no exposure. (b) *Ex-vivo* images (24 h) of IMP301 dependent on FUS exposure. Intratumoral distribution of IMP301 was increased under FUS exposure, compared to no exposure. *FUS* Focused ultrasound, *IMP301* doxorubicin HCl-loaded liposome, *h* hours from IMP301 injection. **Supplementary Table S1.** Body weight of Groups in Study-1. **Supplementary Table S2.** Body weight of Groups in Study-2.

## Data Availability

The datasets used and/or analyzed during the current study are available from the corresponding author on reasonable request.

## Abbreviations

DOX: Doxil
FUS: Focused ultrasound
GEM: Gemcitabine
