# Supplementary material for: Tumor suppression effect of ultrasound-sensitive nanoparticles with focused ultrasound in a pancreas cancer xenograft model
Source: Eur Radiol Exp. 2024 Mar 20;8:39. doi: 10.1186/s41747-024-00436-2 (PMC10951153; doi:10.1186/s41747-024-00436-2)
Supplement: Supplementary file 1 — Additional file 1: Supplementary Fig. S1. (a, b) Cumulative doxorubicin release from IMP301 or Doxil depending on unfocused continuous wave ultrasound irradiation. Both IMP301 and Doxil resulted in enhanced doxorubicin release when irradiated by unfocused ultrasound (continuous wave (100% duty cycle); 29 kHz frequency; 92 W/cm2 intensity). Cumulative release of doxorubicin from IMP301(a) was three-fold greater than that of Doxil (b) when irradiated by unfocused ultrasound. (c, d) Size distribution of liposomes depending on unfocused ultrasound irradiation time. Both IMP301 and Doxil liposomes maintained size throughout the experiment. FUS Focused ultrasound, IMP301 doxorubicin HCl-loaded liposome, US ultrasound. Supplementary Fig. S2. (a) Doxorubicin release from IMP301 or Doxil by FUS exposure. IMP301 released more than two-fold amount of doxorubicin compared to Doxil when irradiated by FUS (2% duty cycle; 1.0-MHz frequency; 2.8 kW/cm2 intensity; 250-Hz pulse repetition frequency; 20 s/spot insonation time). (b) Doxorubicin release from IMP301 depending on the intensity and duty cycle of FUS. When exposed to FUS with same total amount of energy (70.8 W/cm2), doxorubicin release from IMP301 was mainly induced by burst mode of high-intensity beam rather than continuous mode of low-intensity beam. FUS Focused ultrasound, IMP301 doxorubicin HCl-loaded liposome, US ultrasound. Supplementary Fig. S3. (a) Biodistribution of IMP301 dependent on FUS exposure. Increased IMP301 concentration and more selective distribution of IMP301 within the tumor were noted under FUS exposure, compared to no exposure. (b) Ex-vivo images (24 h) of IMP301 dependent on FUS exposure. Intratumoral distribution of IMP301 was increased under FUS exposure, compared to no exposure. FUS Focused ultrasound, IMP301 doxorubicin HCl-loaded liposome, h hours from IMP301 injection. Supplementary Table S1. Body weight of Groups in Study-1. Supplementary Table S2. Body weight of Groups in Study-2. [file 41747_2024_436_MOESM1_ESM.docx]

**Tumor suppression effect of ultrasound-sensitive nanoparticles with focused ultrasound in a pancreas cancer xenograft model**

**ELECTRONIC SUPPLEMENTARY MATERIAL**

**Supplemental Figures**


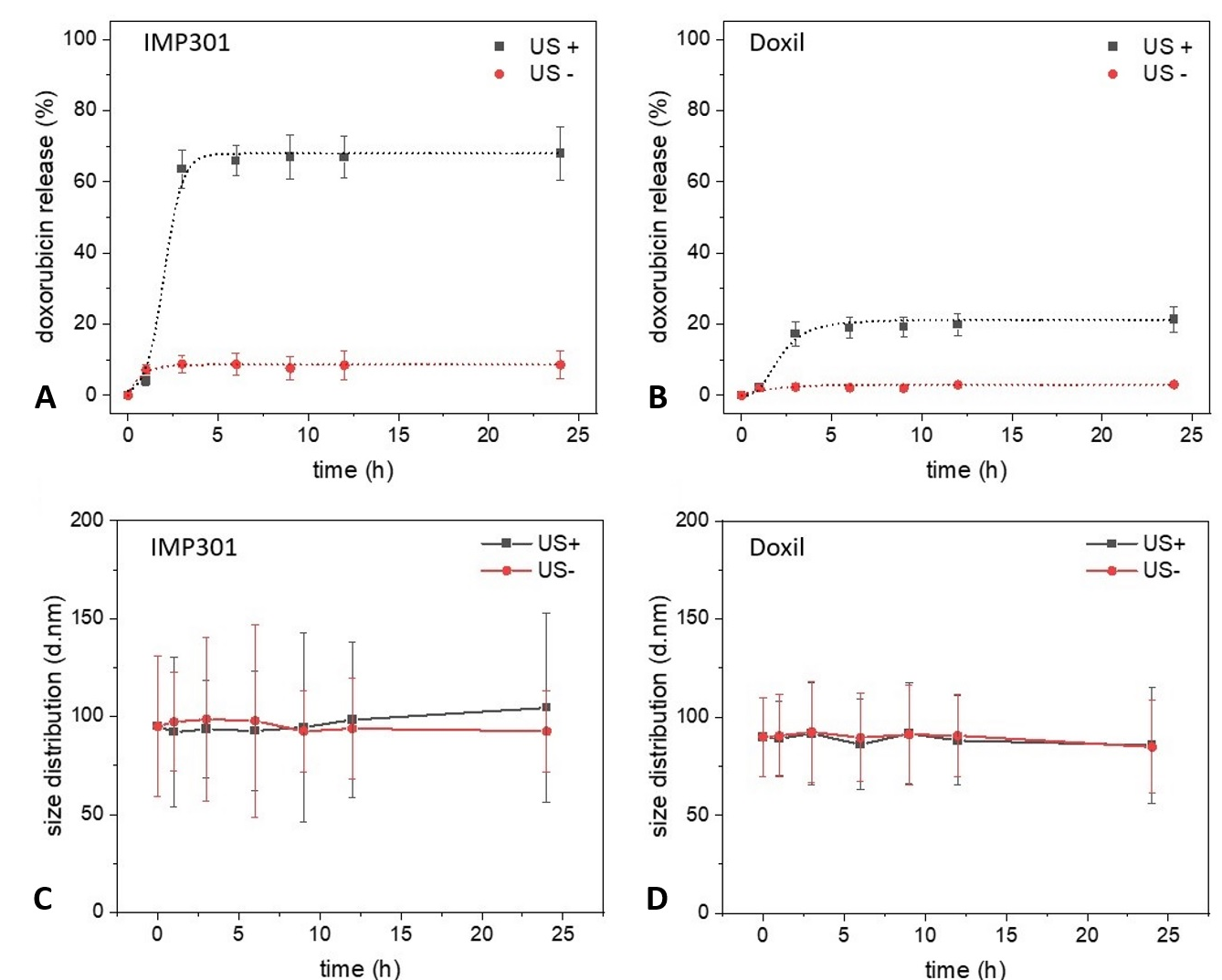


**Supplementary Fig. S1**. (a)(b) Cumulative doxorubicin release from IMP301 or Doxil depending on unfocused continuous wave ultrasound irradiation. Both IMP301 and Doxil resulted in enhanced doxorubicin release when irradiated by unfocused ultrasound (continuous wave (100% duty cycle); 29 kHz frequency; 92W/cm^2^ intensity). Cumulative release of doxorubicin from IMP301(a) was three-fold greater than that of Doxil (b) when irradiated by unfocused ultrasound. (c)(d) Size distribution of liposomes depending on unfocused ultrasound irradiation time. Both IMP301 and Doxil liposomes maintained size throughout the experiment. *FUS* Focused ultrasound, *IMP301* doxorubicin HCl-loaded liposome, *US* ultrasound**.**


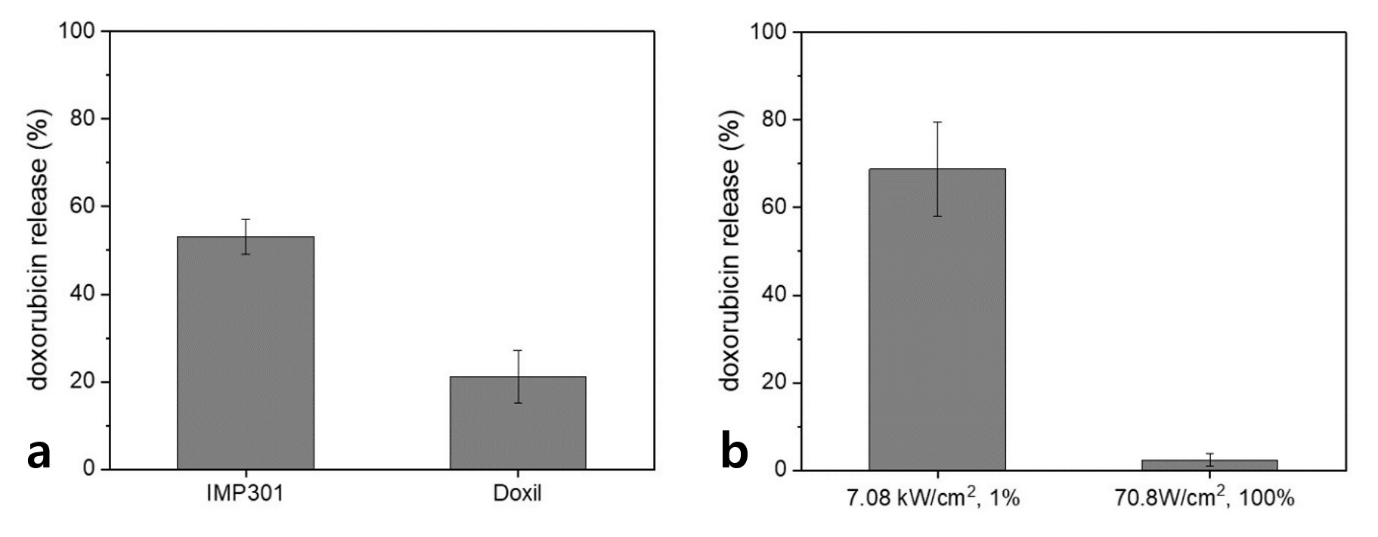


**Supplementary Fig. S2.** (a) Doxorubicin release from IMP301 or Doxil by FUS exposure. IMP301 released more than two-fold amount of doxorubicin compared to Doxil when irradiated by FUS (2% duty cycle; 1.0-MHz frequency; 2.8 kW/cm^2^ intensity; 250-Hz pulse repetition frequency; 20 s/spot insonation time). (b) Doxorubicin release from IMP301 depending on the intensity and duty cycle of FUS. When exposed to FUS with same total amount of energy (70.8 W/cm^2^), doxorubicin release from IMP301 was mainly induced by burst mode of high-intensity beam rather than continuous mode of low-intensity beam. *FUS* Focused ultrasound, *IMP301* doxorubicin HCl-loaded liposome, *US* ultrasound**.**


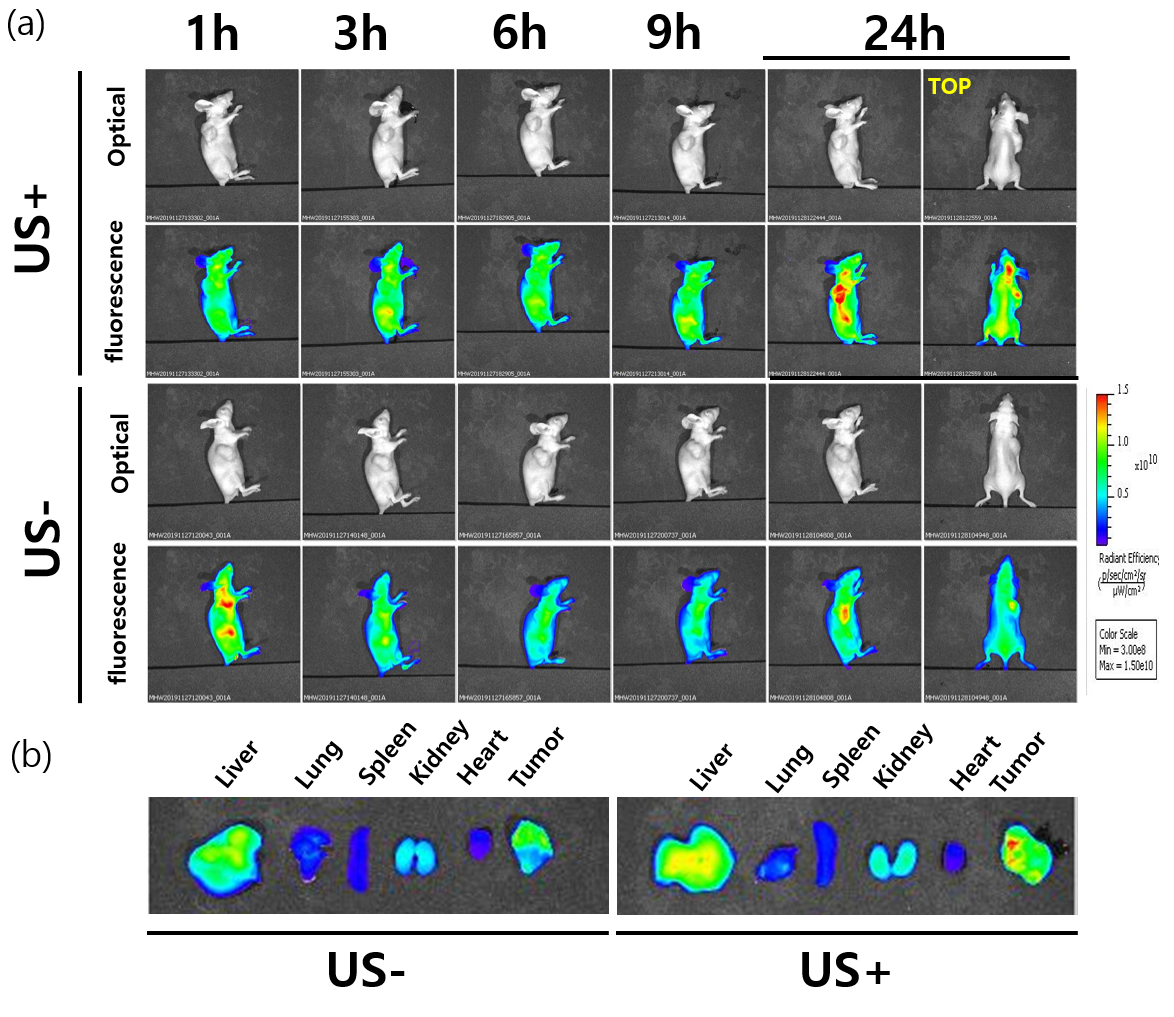


**Supplementary Fig. S3.** (a) Biodistribution of IMP301 dependent on FUS exposure. Increased IMP301 concentration and more selective distribution of IMP301 within the tumor were noted under FUS exposure, compared to no exposure. (b) Ex-vivo images (24hr) of IMP301 dependent on FUS exposure. Intratumoral distribution of IMP301 was increased under FUS exposure, compared to no exposure. *FUS* Focused ultrasound, *IMP301* doxorubicin HCl-loaded liposome, *h* hours from IMP301 injection.

**Supplemental Tables**

**Supplementary Table S1.** Body weight of Groups in Study-1

| Values | Control | GEM only | DOX with FUS | IMP301  (2 mg/kg)  with FUS | IMP301  (4 mg/kg)  with FUS | IMP301  (6 mg/kg)  with FUS | *P-value* |
| --- | --- | --- | --- | --- | --- | --- | --- |
| Weight at 0 week (mg) | 24.2 ± 1.2 | 24.6 ± 1.2 | 24.2 ± 0.7 | 23.2 ± 1.2 | 22.4 ± 0.9 | 22.6 ± 0.7 | > 0.05 |
| Weight at 3^rd^ week (mg) | 25.7 ± 0.9 | 26.8 ± 1.0 | 24.9 ± 0.4 | 25.3 ± 1.3 | 24.6 ± 1.0 | 23.9 ± 0.5 | > 0.05 |
| Weight at 6^th^ week (mg) | 27.1 ± 1.0 | 28.1 ± 1.2 | 26.7 ± 0.3 | 27.1 ± 1.3 | 26.2 ± 1.5 | 25.7 ± 0.4 | > 0.05 |

Numbers indicate the means ± standard deviation. *DOX* Doxil, *FUS* Focused ultrasound, *GEM* Gemcitabine, *IMP301* doxorubicin HCl-loaded liposome.

**Supplementary Table S2.** Body weight of Groups in Study-2

| Values | Control | GEM only | FUS only | DOX with FUS | IMP301  (4 mg/kg)  only | IMP301  (4 mg/kg)  with FUS | *P-value* |
| --- | --- | --- | --- | --- | --- | --- | --- |
| Weight at 0 week (mg) | 24.0 ± 0.6 | 23.7 ± 1.4 | 24.7 ± 1.2 | 23.4 ± 0.7 | 24.6 ± 1.5 | 24.2 ± 1.0 | > 0.05 |
| Weight at 3^rd^ week (mg) | 25.2 ± 1.0 | 24.5 ± 1.4 | 25.6 ± 1.3 | 24.9 ± 0.5 | 25.4 ± 2.1 | 24.8 ± 0.7 | > 0.05 |
| Weight at 6^th^ week (mg) | 25.8 ± 1.0 | 26.4 ± 1.5 | 25.6 ± 1.1 | 25.1 ± 0.9 | 26.0 ± 2.0 | 25.8 ± 0.8 | > 0.05 |

Numbers indicate the means ± standard deviation. *DOX* Doxil, *FUS* Focused ultrasound, *GEM* Gemcitabine, *IMP301* doxorubicin HCl-loaded liposome.
